# A Fully Open-Source Approach to Intelligent Edge Computing: AGILE’s Lesson

**DOI:** 10.3390/s21041309

**Published:** 2021-02-12

**Authors:** Massimo Vecchio, Paolo Azzoni, Andreas Menychtas, Ilias Maglogiannis, Alexander Felfernig

**Affiliations:** 1 OpenIoT Research Unit, Fondazione Bruno Kessler, 38123 Trento, Italy; 2 Eurotech Group, 33020 Amaro, Italy; paolo.azzoni@eurotech.com; 3 BioAssist S.A., 11524 Athens, Greece; amenychtas@bioassist.gr; 4 Department of Digital Systems, University of Piraeus, 18534 Piraeus, Greece; imaglo@unipi.gr; 5 Institute of Software Technology, Graz University of Technology, 8010 Graz, Austria; alexander.felfernig@ist.tugraz.at

**Keywords:** modular approach, IoT, edge computing, open source, open hardware, knowledge-based configuration, recommender systems

## Abstract

In this paper, we describe the main outcomes of AGILE (acronym for “Adaptive Gateways for dIverse muLtiple Environments”), an EU-funded project that recently delivered a modular hardware and software framework conceived to address the fragmented market of embedded, multi-service, adaptive gateways for the Internet of Things (IoT). Its main goal is to provide a low-cost solution capable of supporting proof-of-concept implementations and rapid prototyping methodologies for both consumer and industrial IoT markets. AGILE allows developers to implement and deliver a complete (software and hardware) IoT solution for managing non-IP IoT devices through a multi-service gateway. Moreover, it simplifies the access of startups to the IoT market, not only providing an efficient and cost-effective solution for industries but also allowing end-users to customize and extend it according to their specific requirements. This flexibility is the result of the joint experience of established organizations in the project consortium already promoting the principles of openness, both at the software and hardware levels. We illustrate how the AGILE framework can provide a cost-effective yet solid and highly customizable, technological foundation supporting the configuration, deployment, and assessment of two distinct showcases, namely a quantified self application for individual consumers, and an air pollution monitoring station for industrial settings.

## 1. Introduction

The Internet of Things (IoT) can be described as an extension of the Internet and other network connections to different sensors and devices allowing even everyday objects (the “things”) to have a higher degree of computing, analytical capabilities, and interactions with other objects, online services, and humans [1]. As a concept, it opened a new era of applications and services in several vertical domains. Indeed, since its early infancy, the IoT and its main enabling technologies have been attracting the interest of a multitude of startups and innovative Small-Medium Enterprises (SMEs) willing to enter the IoT market, providing hardware and/or software solutions within several application domains (e.g., home automation, wearables, smart mobility, smart spaces, Industry 4.0) [2].

However, IoT penetration in real-life applications is not happening as fast. According to a recent report by Gartner, it is estimated that 75% of IoT projects will take up to twice as long as originally planned [3]. The main reasons for this delay in IoT real-life application deployment are costs and implementation time. To connect devices and operations with the cloud infrastructure requires repeated fine-tuning. Prototyping an IoT solution is a quite complex process involving the careful selection of the most suitable components, especially when they are produced by third-party vendors that, in most cases, provide specific, closed, and vertical solutions. Thus, too often, to gain control of their own devices and/or the data they collect, users may need to rely on vendor’s backends (through an Application Programming Interface (API)) to connect to proprietary gateways. In other situations, users may need to develop and host their apps on different runtime environments/machines by using some Software Development Kits (SDKs) provided for a specific gateway [4].

Another issue with closed solutions is their cost: at the IoT platform level (i.e., cloud-based solutions for connecting devices to the Internet and for managing them and their data), there is still high fragmentation, without a clear market strategy or adoption [5]. According to the latest IoT Platforms Competitive Landscape & Database 2020, from 450 IoT platform companies in 2017, the market currently counts 620 IoT platform companies, without showing any signs of consolidation [6]. The absence of a standardized way for creating end-to-end IoT applications and of a widely adopted IoT platform model force IoT vendors to implement their own solutions that become vertical, domain-specific, and product-oriented or, in one word, closed. This siloed approach leads to severe privacy and data control issues: devices collect data about end-users’ context and transfer them to external applications hosted on proprietary cloud-based servers. This means that end-users have no full control over their data: indeed, they cannot decide how, when, and what to share, besides not having control over with whom such data are shared (or sold to) [7].

One of the main goals of edge computing is to alleviate the abovementioned challenges by pushing data collection, processing, and reporting functionalities as close as possible to end-users. IoT and edge computing are currently playing a key role in the European digital strategy for the future. Recently, the European Commission (EC) identified some specific strategic priorities for a “Europe fit for the Digital Age” to, quoting its President-elect’s words, “ensure that Europe fully grasps the potential of the digital age and strengthens its industry and innovation capacity” (https://ec.europa.eu/commission/commissioners/sites/comm-cwt2019/files/commissioner_mission_letters/mission-letter-margrethe-vestager_2019_en.pdf, accessed on 12 February 2021). Indeed, investing in IoT and edge computing today represents a keystone for the “Digital Age”, as they represent technology game-changers in the digitalization process and because they are enablers for other technologies such as big data, Artificial Intelligence (AI), and cloud computing. Since 2015, the EC has been focusing research and innovation efforts on edge computing, with specific calls for proposals: in this fertile ground, the AGILE (“Adaptive Gateways for dIverse muLtiple Environments”) project started playing an active role within the European transition towards the coveted digital age, as it anticipated that many of the hot research topics today have not been completely solved yet. To confirm this, the 2021 European Strategic Research and Innovation Agenda for the Electronic Components and Systems dedicated a whole chapter to edge computing, edge AI, and advanced control, hence corroborating the key role played by such technologies during the transition towards the European digital age [8].

Fully aligned with the EU vision of the edge computing paradigm, the AGILE project developed a generic, low-cost, multi-purpose, and adaptive framework for IoT gateways, capable of integrating various types of devices (wearables, home appliances, sensors, and actuators, etc.), enforcing fine-grained access control and data-retention policies. The modularity includes connectivity both for field and wide area network communications (e.g., Bluetooth Low Energy, ZigBee, ZWave, 433 and 866 MHz RF, LoRa, etc.).

**Figure 1:**
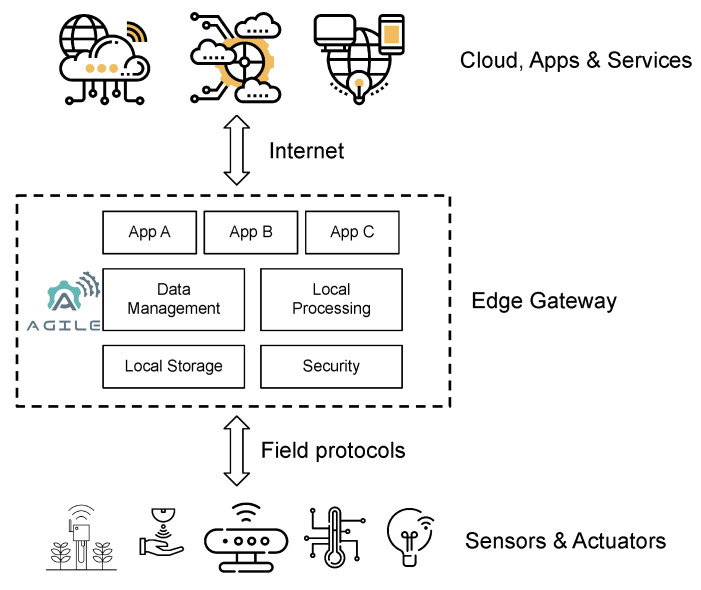
Logical view of edge computing, the AGILE (“Adaptive Gateways for dIverse muLtiple Environments”) gateway, and its main functionalities.

As depicted in Figure 1, the proposed framework is positioned between the cloud app/service layer and the sensor/actuator one, supporting not only the interconnection between various open and private cloud environments but also data management, local storage, processing, and device control functionalities directly at the local gateway level, hence enforcing security and privacy. More specifically, with the main goal of hiding the technology complexity behind an IoT system, the AGILE project delivered two distinct versions of the hardware gateway, namely the maker’s version (based on the popular RaspberryPi platform for easily prototyping of IoT solutions) and the industrial version (based on the existing Eurotech M2M gateway family). The objective of this Research and Innovation Project (RIA) was to enable users and developers to easily install IoT applications that run on the gateway and to have full control over management of the connected devices, processing of data, as well as communication with external services. The project created an ecosystem of IoT applications that can be shared and recommended between users and developers by leveraging existing initiatives of key stakeholders and communities, such as Docker, the leading technology for software containerization [9]. In this way, users can search, install, manage, and securely share IoT apps through the Docker app marketplace, developed within the project.

Looking at the most recent research literature related to edge computing, a fresh survey reveals that, according to Google Scholar, in 2015 (i.e., when the AGILE project proposal was written and submitted to the EC portal for evaluation), the number of papers related to “edge computing” was less than 400. Then, from 2015 to 2018, this number grew tenfold, hence entering the rapid growth period and estimating around 7000 papers in 2019 [10]. Only in 2018 was a comprehensive survey of the most promising research-oriented edge computing platforms conducted, in which the authors of [11] compared the most mature solutions at that time in terms of the type of nodes (constrained hardware, full-fledged nodes, etc.) and type of networks supported (3G, Wi-Fi, etc.), thus focusing on the infrastructure level. The golden age of edge computing also affected the industry, and as a consequence, several innovation projects (from open-source and community-based to co-funded and commercial initiatives) started proliferating globally. It is not a coincidence that edge computing reached the peak of Gartner’s Hype Cycle for Emerging Technologies in 2017 [12]. However, a comprehensive survey of all such initiatives is out of the scope of this paper, also considering that a pertinent and very detailed comparative analysis has been recently published by one of the coauthors of this paper [13]. Briefly, this document provides a global overview of the IoT market, analyzing both technical and application areas, identifying trends and industry, and comparing the achievements of more than one hundred research projects revolving around the intersection of IoT and edge computing. We invite the interested reader to refer to [10,11,13] and the references therein for more details.

In this paper, we summarize two of the main features offered by the AGILE framework, namely its flexibility and reconfigurability. As we will see in Section 2, these features, combined with its software and hardware openness, make the proposed framework particularly attractive for small to medium enterprises, since it can provide a cost-effective yet solid and highly customizable technological foundation supporting the configuration, deployment, and execution of end-to-end applications in various domains. Table 1 visually maps some of the most mature edge computing solutions currently available in terms of such features, enriched with our critical assessment of their technology maturity level (high/medium/low). Due to space limitation, this summary cannot be comprehensive; therefore we have limited our selection to the most mature alternatives, namely Azure IoT Edge (the Microsoft solution to edge computing), AWS IoT Greengrass (the Amazon solution to edge computing), and EdgeX Foundry (the Linux Foundation solution to edge computing). For a more extensive survey and comparative analysis, we invite the interested reader to refer to [14].

The remainder of this paper is structured as follows: in Section 2, we present two of the unique features of the AGILE IoT gateway, namely its flexibility and reconfiguration capabilities [15]; Section 3 introduces two orthogonal scenarios where the AGILE gateway can be configured, deployed, and assessed, namely a consumer scenario realizing a Quantified Self (QS—the cultural phenomenon of self-tracking with ICT technology [16]) application and an Air Pollution Monitoring Station (APMS) for industrial settings [1]. Then, in Section 4 the presented showcases are evaluated, deriving useful best practises and the main lessons learned, while in Section 5, we draw our conclusions.

## 2. AGILE’s Unique Features

The AGILE IoT gateway presents several figures of merit, but due to space limitations, we cannot describe all of them. In this section, we focus on two unique features characterizing the proposed framework, namely its flexibility and reconfigurability.

### 2.1. A Flexible and Modular Solution for a Dynamic and Demanding Market

The market of multi-service IoT gateways is extremely dynamic, and from customers’ and vertical applications’ point of view, it is characterized by small/medium volumes of highly customized gateways. Small volumes, high customization, and low prices represent diverging factors, making the identification and design of a suitable solution very difficult (often impossible). This is particularly true for standard monolithic hardware, which requires a new design for potentially every customer or vertical application [17].

To address a similar dynamic and demanding market, the AGILE platform offers an open, modular, flexible, and reusable IoT solution, easily adaptable to different contexts and domains [18]. Hardware modularity and reuse, supported by an appropriate design methodology (called Design for Modularity (DFM)) and by the use of modules relying on standard interfaces (e.g., COMExpress and HAT specification), allow a user to find the right trade-off between volumes, customization level, price, and time-to-market. Furthermore, the configurable architecture of an AGILE gateway is based on standard hardware and software technologies, simplifying the gateway partitioning into modules, promoting reuse, and improving the time-to-market: even when a customer requires a single board solution to contain as much as possible the price, the flexibility of DFM allows a user to consolidate the AGILE reference design in a single board solution, with no modularity at all.

**Figure 2:**
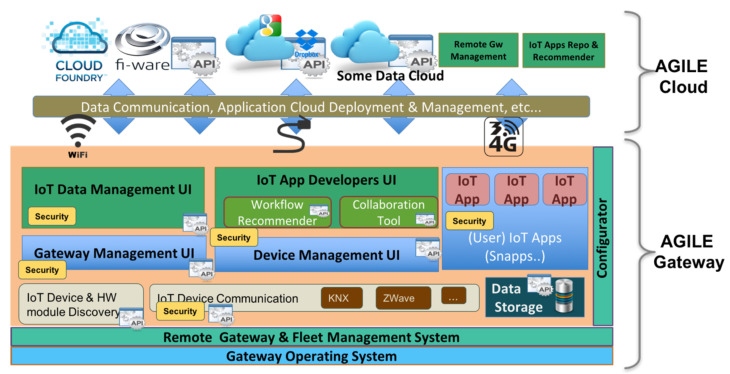
The AGILE modular software stack.

The software modularity of the AGILE platform ensures flexibility and a high level of customization for the AGILE software stack and for vertical application: modularity and customizability are designed as an integral part of the development process, from the selection of the underlying operating system and deployment model to the selection and eventual development of individual software components [19]. Software modularity includes hardware abstraction of each hardware module; field connectivity; local and wide area network connectivity; security and privacy; components to enable the remote management of the gateway; edge processing; local storage; cloud platforms integration; and even visual tools to simplify application development, gateway integration, and usage (see Figure 2).

The AGILE solution was adopted and evaluated in five different pilots (focused on health care, cattle monitoring, environmental monitoring, and enhanced retail and port area monitoring) and 27 projects developed by external partners (through a cascade funding scheme) focusing on various vertical application domains, such as smart city, connected buildings, smart energy, health care, smart agriculture, smart retail, Industry 4.0, and even education. This large set of use cases demonstrated that the AGILE solution can efficiently satisfy multi-sectorial requirements, supporting heterogeneous vertical applications. This adaptability was possible only by reusing the hardware and software modules to compose an IoT gateway specifically conceived for a particular use case and by relying on AGILE APIs, SDK, and code examples to implement the use case-specific business logic.

### 2.2. Re-Configurability and Recommending Capabilities

A key issue in successfully developing and deploying an IoT infrastructure is the provision of intelligent and highly efficient techniques and algorithms that support i) the ramp-up of complex systems and ii) their administration and usage at runtime. The first challenge can be tackled based on efficient and personalized configuration technologies [20,21] that help make ramp-up processes structured and the outcome consistent. The second challenge can be tackled with recommendation technologies that help predict relevant items and suggest parameter settings to users (both the designers of an IoT system and the end-users who use the specific IoT solution) [22].

AGILE technologies include efficient and personalized configuration features supporting the declarative modeling of highly variant and complex IoT infrastructures (e.g., in terms of Answer Set Programs (ASP) [23] and Constraint Satisfaction Problems (CSPs) [24]). The main innovations in this context are (i) the ability of the developed configurators to adapt to new scenarios by exploiting installation preferences of similar customers and (ii) the ability to determine solutions in an efficient and personalized fashion, based on learning problem-specific search heuristics [20]. It is often the case that some user-defined requirements are inconsistent. In such contexts, AGILE provides automated analysis operations in terms of model-based diagnosis. Such approaches help to identify minimal adaptations of requirements in a way that at least one solution can be identified [21].

The administration and usage of complex IoT infrastructures must be supported by recommendation technologies [20] that help designers and developers not only figure out the relevant sensors and applications needed for a specific customer but also support end-users in various ways. For instance, as we will describe in the Quantified Self showcase, users receive recommendations regarding their eating behaviors and different possibilities to improve their physical fitness. Such recommendations can be partially determined based on other “success stories”, i.e., data (in an anonymized form) about users who managed to significantly improve their physical fitness and their eating behavior. Basic recommendation approaches that can be applied in this context are collaborative filtering (CF) and content-based filtering (CBF). Using CF, recommendations are determined based on “word of mouth” promotion, and users with similar preferences and behavior (so-called nearest neighbors) are the basis for determining recommendations for the current user. CBF is based on the idea of “stable preferences”, for example, since a user liked a specific menu recommendation in the past, similar menus are recommended in the future. For further details on existing recommender systems approaches, we refer the reader to [25,26].

## 3. AGILE Showcase

In this section, we present two orthogonal use cases selected among the 32 use cases developed during the project execution: a consumer use case and an industrial application.

### 3.1. Consumer Use Case

**Figure 3:**
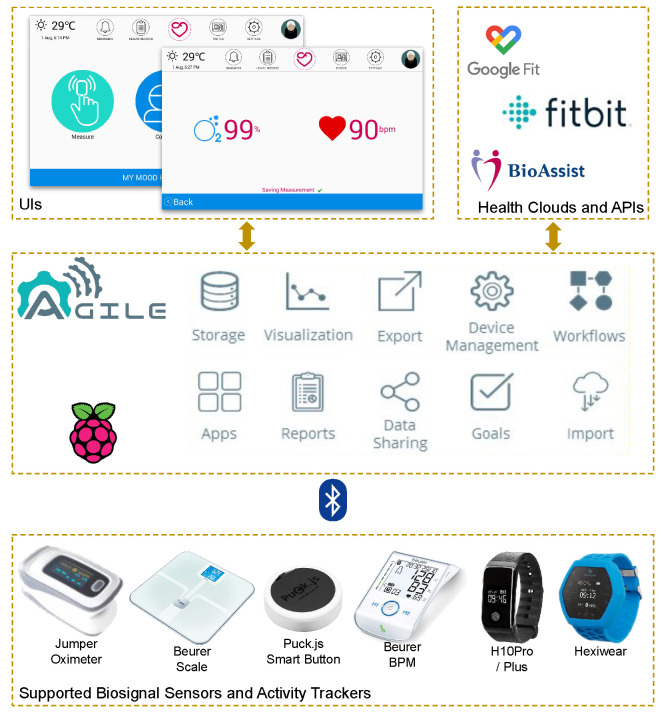
The Quantified Self application.

For assessing the usability and the effectiveness of the AGILE framework in a home environment, a mobile health application was proposed. This application targets data acquisition on aspects of a person’s daily life through a modern platform that eliminates the need for additional applications or hardware. This is the concept of Quantified Self (QS), depicted in Figure 3 together with the respective web application running on top of the gateway. The latter aggregates data from the other components, presenting them to the user in various forms and allowing him/her to set goals and to follow the progress towards such goals [27]. Wearable activity trackers and medical sensors automatically communicate with the gateway as soon as they are within the communication range of each other to offload the most recent collected data. The application is accessible through a state-of-the-art web user interface that communicates with the gateway components in the background to perform various operations. These operations span from the registration of new sensors and authentication to remote cloud platforms (e.g., Fitbit, Google Fit), visualization, and reporting of the acquired data. Users can visualize and manage their data, create reports, and export the data from the gateway or even import past data from other cloud sources. Furthermore, based on the specific goals for each user and the collected data, the application produces personalized messages and recommendations [28].

The AGILE framework implements the functionality required to collect data from user-owned peripheral devices and cloud-based platforms, to store data locally, and to provide data visualization and processing to gain useful insights. The deployment of the AGILE software was carried out using the Balena infrastructure, which also enabled the following features:

- remote multi-container software deployment to devices,
- monitoring of device status and container error conditions during application development,
- monitoring of software deployment progress,
- use of Balena’s supervisor API to provide information on local and remote IP addresses to the user and to control/restart the device, and
- the setting of environmental variables in the device to enable advanced features of the AGILE software stack.

One key feature of the QS application is the provision of personalized recommendations to end-users by analyzing the user activity data stored locally in the gateway. In this context, three different approaches were examined:

- Virtual Coach: to motivate subscribers/users to perform sports activity, the Virtual Coach collects their demographic information (age, location, physical condition, medical history, chronic diseases, etc.). A recommender engine then calculates similarities among users based on their demographic data. Using similar users’ information, new activity plans (how often, what to measure, which activities, etc.) as well as new IoT devices (wristbands, step counter watches, etc.) can be recommended to users.
- Virtual Nurse: the Virtual Nurse motivates different types of chronic patients (diabetes, asthma, cancer, cardiovascular, etc.) to reach their goals based on a recommended plan. It collects the measured data of patients and checks their health condition targets. If the measured and target values are too far apart, then personalized recommendations can be provided to such patients.
- Virtual Sleep Regulator: the Virtual Sleep Regulator helps insomnia patients improve their sleep quality. It uses collaborative filtering techniques to recommend an appropriate waking/sleeping plan for the patients. Chronic insomnia (defined as the difficulty initiating or maintaining sleep, awakening too early in the morning, or non-restorative sleep) is the most common sleep disorder among adults.

### 3.2. Industrial Case

The industrial version of the gateway introduces a new generation of fully modular embedded systems, evolving towards a global, flexible, standardized hardware capable of satisfying, with its high level of configurability, completely different applications in different vertical domains (transportation, industrial, environmental monitoring, medical, logistics, security, surveillance, etc.). In the industrial gateway, every main architectural element is a module: CPU, carrier, I/O, internal expansion, external expansion, storage, power supply, and gateway enclosure. A module represents the minimal standardized building block and, depending on the application, can be a commercial module, an AGILE standard module, or even a custom module [29]. This approach, called “Design for modularity” (DFM), follows and extends an emerging trend in hardware design and manufacturing known as “Build to Order” (BTO). With BTO, the product manufacturing process starts only after a customer’s order is received and, only when the order is confirmed, a pull-type supply chain operation starts. BTO is used to create highly customized products, but only when required, allowing flexible design processes, reducing inventory, simplifying the supply chain, and keeping manufacturing costs at bay.

The DFM complements and extends BTO to the design and manufacturing phases, allowing us to reduce the effects of fixed and development costs and to provide custom products at a lower price. The combination of DFM and BTO allows us to create custom designs from a “library” of reusable modules, and the lower cost of reuse compensates for the high costs typically associated with low-volume custom products [9,18].

The modularity is based on three categories of hardware modules:

1. logical modules: these modules are design-time modules and disappear in the final implementation of the gateway;
2. integrated modules: these modules are design-time modules and persist in a modular form also when integrated into the gateway; and
3. physical modules: these modules become real physical modules.

The DFM is organized into two phases:

1. definition of a reference design: starting from the analysis of the company expertise, vertical markets, customers, profile, and needs, a set of general requirements is identified and the gateway architecture is partitioned into modules. Subsequent refinements based on technical aspects, manufacturing processes, stocking planning, operational aspects, vertical application evaluation, and costs balancing allow for the definition of the reference design of a general-purpose modular gateway. This design could be directly implemented but is extremely more useful as a reference model [30].
2. definition of a vertical consolidated design: starting from the reference design, the analysis of the customer/application requirements allows us to select the subset of modules strictly required for that customer/application. Hence, the reference design is consolidated in a custom gateway and the consolidation process exploits as much as possible the modularity of the reference design, trying to minimize the use of custom modules.

**Figure 4:**
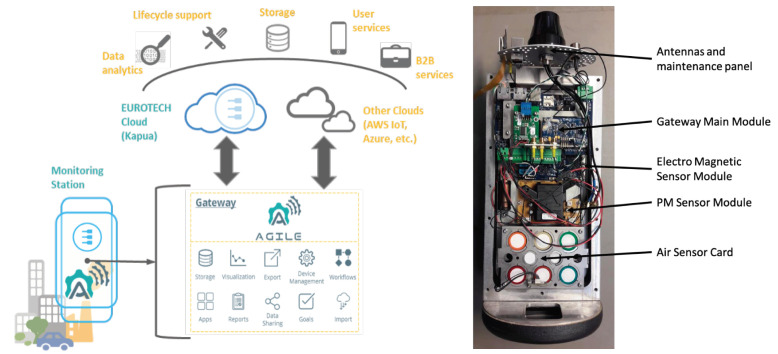
The industrial pilot architecture and the prototype of the Air Pollution Monitoring Station (APMS).

Adopting the DFM, the reference design of the AGILE industrial gateway was consolidated in a modular Air-quality and Pollution Monitoring Station (APMS) that adopts the AGILE modular software stack. Environmental pollution has become an issue of serious international concern and is increasingly stimulating the development and adoption of solutions to monitor, prevent, and reduce the effects of pollution. This challenging domain has an important economic and societal impact and is characterized by a long history of monitoring methodologies and technological solutions, unfortunately, characterized by high development and maintenance costs, low territorial coverage, and complex certifications. These limitations have confined the diffusion of high-end monitoring solutions to a limited set of vertical contexts, typically managed by public authorities. AGILE proposed a low-cost solution based on multiple APMSs distributed in a wide area and responsible for providing multi-modal, multi-source, certifiable, and pervasive monitoring of air quality and pollution levels. The environmental information was collected and processed locally by the APMSs and published on the cloud, where it becomes easily accessible to the final users, B2B services, mobile, and enterprise applications (see Figure 4).

The main benefit of the proposed solution is the possibility to deploy a pervasive network of low-cost APMSs capable of providing high-quality and certifiable data acquisition, with a rich set of environmental parameters. The large amount of data collected represents a valuable asset for new added-value services that can generate new business opportunities.

**Figure 5:**
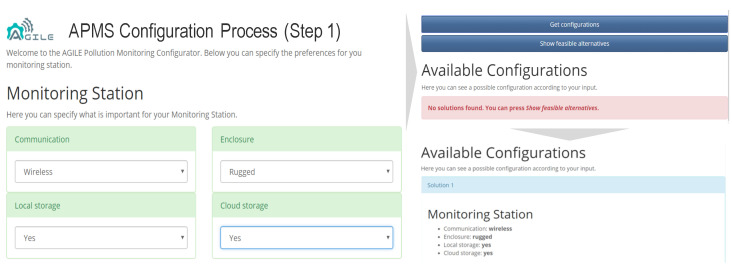
The APMS configurator.

The DFM allows us to identify the hardware configuration that better satisfies the required features and the price point of a specific environmental monitoring application. The requirements provided by the customer drive the selection of the hardware modules, without preventing future extensions of the monitoring station. The reference design was customized to develop a new low-price device characterized by smaller size, higher integration, lower power consumption, and modularity focused only on sensing and connectivity. Then, to further simplify the selection of sensing and connectivity modules, a specific ramp-up configurator was also developed (see Figure 5 [9]). Starting from a description of the deployment environment and the specific environmental application, the configurator automatically calculates the best modules for the APMS. The configurator is based on a flexible knowledge representation of the APMS configurations and provides efficient reasoning for solving configuration problems, supporting the operator with diagnostic information when inconsistencies in the configuration are identified.

From a software perspective, the APMS adopts an open-source and modular AGILE software stack (including AGILE customization of the Eclipse Kura framework, which valorizes the modular hardware; simplifies its use and management; and provides tools, services, and API that can simplify the integration of existing systems and the implementation of use case-specific business logics. The IoT cloud platform adopted as a counterpart of the AGILE software gateway was Eclipse Kapua, a modular, integrated, interoperable solution to manage and integrate a fleet of APMS.

During the last six months of the project, Eurotech decided to start engineering APMS prototypes to create a new product line focused on environmental monitoring. After one year, when the certification process concluded, a new product was released with the name “ReliaSense” (https://www.eurotech.com/en/products/intelligent-sensors/environmental-monitoring-systems/reliasens-19-15, accessed on 12 February 2021). Moreover, considering the positive experience of ReliaSense, the reference design was adopted for the engineering of a second product line focused on the transportation market: the BoltGate family of embedded units (https://www.eurotech.com/en/products/subsystems/embedded-computers/boltgate-20-25, accessed on 12 February 2021). This important result confirmed the flexibility of AGILE and of the DFM, which allowed us to address completely different markets starting from the same reference design.

## 4. Lessons Learned

In this section, we report the final evaluation of the AGILE platform in the form of the main lessons learned deriving from the individual showcases.

### 4.1. Consumer Use Case

The design, deployment, and operations of the QS application on top of the AGILE framework were evaluated in a real-life environment. Specifically, the evaluation was related to the core functionality of the gateway as well as concerning the specific sensors that should be used and development of the application itself.

The first important finding was that selecting suitable sensor devices with a reliable access API was challenging due to the rapid release of new devices that have to be launched on the market. Additionally, several companies that developed such products did not readily release the access API if there was not a compelling business case and due to fear of loss of intellectual property in front of their competitors. At the beginning of the project, we used a Hexiwear (https://www.mikroe.com/hexiwear, accessed on 12 February 2021) device for activity measurements, but this could not be used with actual users since its mechanical structure was not robust enough for real use. The selected device that we used came with its own sets of issues and a proprietary API that needed a custom initialization sequence and changes to the driver API to accommodate the transmission of ad hoc commands.

Moreover, trying to use a generic API to access Bluetooth Low Energy (BLE) devices with widely differing access characteristics proved to be equally challenging. The application required an ad hoc functionality (i.e., checking if two devices are within their communication range and start beaconing), while the open-source libraries needed to provide APIs to common languages such as Java and Node.js did not have the required stability necessary to provide stable communication in a production environment. The existing DBus (https://packages.debian.org/stable/dbus, accessed on 12 February 2021) protocol implementations had several incompatibilities that prevented full interoperability between devices.

Regarding connectivity to the AGILE gateway, there are also some lessons learned: due to the widespread use of Network Address Translation (NAT) technologies, a variety of methods exist to give user-friendly names to devices. Unfortunately, these methods are not universally supported by the home routers, hence complicating the initial setup of the gateway. We used a variety of available applications to scan local networks to find the IP address of a gateway to implement the initial configuration.

From the end-user perspective, the added value of the solution includes the following aspects:

- a fully automated solution requiring minimum engagement from end-users;
- improvement of the health and well-being of end-users;
- the motivation of users to start social, physical, and self-caring activities;
- low cost; and
- enhanced security and privacy, through a local storage policy of collected data.

### 4.2. Industrial Use Case

The device-to-cloud approach adopted in the industrial use case was demonstrated to be extremely efficient and well suited for the domain of environmental conditions monitoring. During the operations, the APMS and Eclipse Kapua cooperated seamlessly, providing a very good solution for data acquisition, local processing, transmission, storage, and fleet remote management for the entire product life cycle.

During the requirement analysis in vertical application, the configurator simplifies and optimizes the selection of the features that must be available in the APMS, and starting from this selection, the hardware modularity ensures the final availability of the best APMS, both in terms of costs and functionalities. The deployment of the APMS is fully supported by AGILE Kura functionalities, such as certificate management, geo-localization, data flow design, remote configuration, and remote management. Remote management is fundamental for the operation phase, providing full control in the APMS, from sensor configuration to process control, data flow management, data acquisition management, software updates, etc. Software updates are managed by the provisioning feature of AGILE Kura, which includes the possibility to update over the air the firmware of the APMS. Finally, remote management and certificate management simplify and improve the security of the APMS retirement at the product’s end of life.

During the deployment phase, configuration of the APMS was stored on the cloud, but this approach revealed several practical issues related to the sensor and algorithm calibration. The calibration process requires precision that is ensured in the laboratory environment where it is performed: the calibration consists in the cyclical process of exposing the APMS to controlled environmental conditions (e.g., a defined percentage of CO dissolved in the air), checking the data collected from sensors, storing it on the cloud, and finally tuning coherently the sensors and/or algorithms parameters. This cyclical process is concluded when the data detected by the sensor matches the actual physical quantity to be measured. In this process, time could become a critical issue, specifically in a real application involving hundreds of APMSs: in the laboratory set-up, every controlled change in the physical quantity measured requires around 1 minute to appear on the cloud side, and this delay affects all the calibration processes, for every single sensor of all the APMSs of the fleet. These delays highlight that the device-to-cloud approach is not the most efficient solution for calibration, testing, and debuggin of sensors and algorithms.

A first solution to avoid this inefficiency consists of working with the gateway local database, through the AGILE local web interface, to avoid the connectivity delays and to significantly speed-up the calibration and test processes. However, the cloud integration platform provides a smarter way to reduce deployment and maintenance times/costs. Kapua remote-control functionalities allow us to remotely calibrate large-scale deployments: the first APMS of the fleet is calibrated in the laboratory environment; it is deployed; and subsequently, it is used as the “reference sample” for the calibration of the entire fleet. Each APMS of the fleet is temporarily installed very close to the reference APMS and remotely calibrated. The calibration process is still affected by the delays introduced by the IoT infrastructure but, being performed directly on the field (not in a laboratory), can be carried out remotely by a single operator in parallel on multiple APMSs. Deployment that typically requires one week can be reduced to one day, with a significant reduction in certification and deployment costs.

## 5. Conclusions

Edge computing, in its most general meaning, pushes memory and computational power out of traditional data centers, getting them as close as possible to the location where they are needed. Often, this means personal devices or everyday home appliances, hence realizing the original vision of the consumer IoT [28]. However, it can also mean advanced industrial equipment or, more generally, physical units that are distributed across different industrial IoT factories in the future [31]. Moreover, besides pure technological aspects, it means also consolidating and widening the professional skills of the people involved in this engineering area [13]. In this arena, several hardware, software, and end-to-end technology solutions have been proposed so far for supporting such a distributed computing paradigm.

In this paper, we presented AGILE: a generic, low-cost, multi-purpose, and adaptive IoT gateway framework able to intelligently accommodate various types of devices, communication protocols, and networking interfaces and technologies. To show its features and capabilities, we resorted to a detailed description of two showcases, selected among more than 30 projects developed during the project execution by project partners and external adopters. However, AGILE represents only the first positive trial of a much wider spectrum of research activities: it offers a solid starting point to further investigate hardware and software modularity, increased interoperability, smarter recommendation technologies, industry-grade remote control and end-to-end security solutions. We believe that these focus areas represent promising research directions, and, alongside them, we have already started several public/private programs. To conclude this paper, in the following, we will briefly summarize them.

Regarding hardware modularity, the current methodology is largely based on COMExpress bus, a solution that ensures high-speed connectivity, a small form factor, and compatibility with a large market of existing modules. This solution is perfectly tailored for an embedded system, but we are investigating new architectures based on module stacking and flipping, which could optimize the adoption of this interface in term of compactness, module reuse, performance, and power consumption. On the software side, modularity is based on container technologies, and in order to ensure gateway evolvability [32], we are investigating other solutions that improve cross-platform compatibility, increase application performances such as if it does not run in a container, allow a wider range of application (e.g., an app with a graphical User Interface, UI) and provide a better native support for security [33].

A higher level of interoperability among different devices connected either to the same IoT gateway or through independently developed systems to the same IoT infrastructure represents a key factor for the uptake of the global IoT market. In this domain, we are focusing our research and innovation efforts on solutions capable of ensuring interoperability through automatic protocol translation [34]. This approach should mitigate some of the major issues of traditional approaches based either on simple hard-coded protocol gateways or on more complex adapters, proxies, and middlewares characterized by low scalability and high maintenance costs. Regarding recommendation technologies, the outcome of a recommendation algorithm is, in many cases, based on limited explanations [20]. For instance, it is based on the preferences of similar users, while more complex explanation approaches could be developed. As an example, one related branch of research is to combine machine-learning-based recommendation approaches with knowledge-based ones and to exploit semantic knowledge for the generation of deeper explanations. Moreover, an open issue in the context of synthesis (configuration) and analysis (diagnosis) operations is scalability: in this context, algorithmic approaches able to fully exploit the capabilities of existing parallel architectures have to be developed. In this case, the goal could be to enable the development of algorithms based on speculative programming [35].

Regarding the control and management of large fleet of gateways, further research and engineering efforts have to be focused on more efficient command and control protocols, on the development of industry-grade embedded brokers for telemetry and on the consolidation of the cloud platform that allows for the control and management of an entire fleet.

Finally, though several security aspects have been successfully addressed during the project execution [36,37], due to the project focus, there is still research and development effort to expend. End-to-end security represents a fundamental aspect to ensure trustworthiness and to improve user acceptance. Indeed, starting from simple authentication and communication encryption, we are investigating secure hypervisor solutions, double-authentication strategies, more solid data encryption algorithms, firewall-friendly communication strategies, and cloud-level security mechanisms, keeping in mind remotely supporting large fleets of deployed gateways. 

## Figures and Tables

**Table 1:** A visual comparison of the most mature edge computing solutions in terms of their flexibility, reconfigurability, openness, and maturity level.

	Flexibility and Modularity	Reconfigurability and Recommending Capabilities	Software and Hardware Openness	Maturity Level (Low/Medium/High)
Azure IoT Edge	✓	✓	✗	high
AWS IoT Greengrass	✓	✓	✗	high
EdgeX Foundry	✓	✗	✓	medium
AGILE	✓	✓	✓	medium
